# Subretinal Drusenoid Deposits in a Patient With HELLP (Hemolysis, Elevated Liver Enzymes, Low Platelet) Syndrome

**DOI:** 10.7759/cureus.52239

**Published:** 2024-01-14

**Authors:** Ceren Durmaz Engin, Omer Karti, Kutlay Kandemir

**Affiliations:** 1 Department of Ophthalmology, Buca Seyfi Demirsoy Education and Research Hospital, Izmir, TUR; 2 Department of Ophthalmology, Faculty of Medicine, Democracy University, Izmir, TUR

**Keywords:** subretinal deposit, subretinal drusenoid deposit, preeclampsia, malignant hypertension, hellp syndrome

## Abstract

Subretinal drusenoid deposits (SDD) are findings that can be observed in age-related macular degeneration as well as in ischemic ocular diseases. These deposits are believed to be of prognostic importance, as they have been shown to be associated with choroidal neovascularization. HELLP (hemolysis, elevated liver enzymes, low platelet) syndrome is a condition linked with severe preeclampsia, and it presents ocular findings such as hypertensive retinopathy, serous retinal detachment, and cortical visual impairment. This case report discusses the presence and course of SDD in a female patient who presented with hypertensive retinochoroidopathy secondary to HELLP syndrome.

## Introduction

Subretinal drusenoid deposits (SDD) are granular extracellular deposits on the top of the retina pigment epithelium (RPE) and typically have a slightly conical appearance in optical coherence tomography (OCT) compared to regular drusen [1]. The impairment of choroidal circulation, especially choriocapillaris ischemia, is proposed as the mechanism for their development. SDD have been shown prospectively to be associated with essentially double the rate of onset of both choroidal neovascularization and geographic atrophy compared with soft drusen in age-related macular degeneration (AMD) in the older population [2]. Although most commonly related to AMD, the association of SDD with ischemic ocular conditions secondary to systemic vascular disorders, including stroke, coronary artery disease, and cardiac failure, was documented [3].

The HELLP syndrome is a pregnancy-related condition, and the abbreviation stands for hemolysis, elevated liver enzymes, and low platelet count. It is typically considered a subtype of preeclampsia, which is characterized by the onset of elevated blood pressure in the second half of pregnancy. The association of HELLP syndrome with hypertensive retinopathy (16% of all cases), serous retinal detachment (SRD) (3.7%), and cortical blindness (2.7%) was documented in a previous study [4]. In this report, we describe a case of bilateral SDD in a patient with HELLP syndrome secondary to hypertensive retinochoroidopathy.

## Case presentation

**Figure 1:**
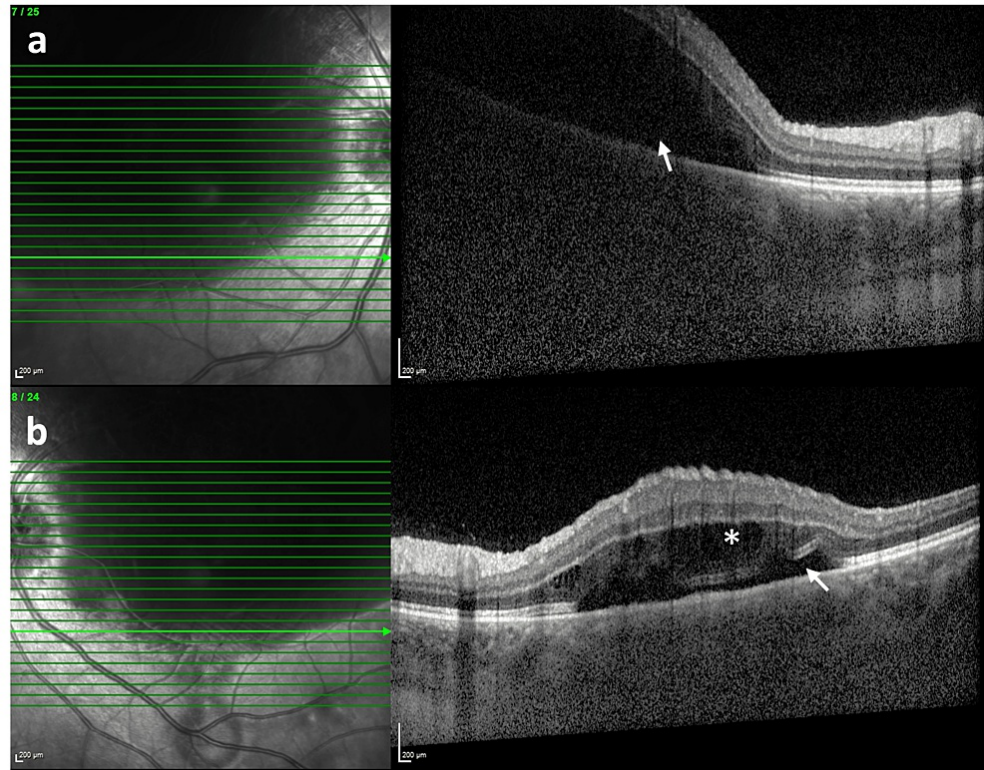
Spectral domain optical coherence tomography scan at first presentation The horizontal SD-OCT scan demonstrates marked serous retinal detachment (white arrow) in the right eye (a) and a shallow serous detachment (white arrow) with intraretinal cysts (asterisk) in the left eye (b). The extent of the lesion is partially visible in the infrared reflectance image in both eyes. SD-OCT, Spectral domain optical coherence tomography

A 17-year-old primigravida woman with limited prenatal care presented to the emergency department with vaginal bleeding, acute onset of severe headache, and bilateral vision loss at 28 weeks of gestation. She had no history of pre-existing medical conditions, pharmacotherapy, or recreational drug use. Her blood pressure was 190/110 mmHg on presentation. She consulted our department, and her ophthalmologic examination revealed a best corrected visual acuity (BCVA) of 0.1 in oculus dexter (OD) and 0.4 in oculus sinister (OS) with a Snellen chart (converted to decimals) along with normal anterior segment findings and intraocular pressures of 14 mmHg bilaterally. There was no relative afferent pupillary defect (RAPD). Dilated fundus evaluation revealed an optic disc edema and a massive serous retinal detachment (SRD) area, which was also documented in spectral domain optical coherence tomography (SD-OCT) along with intraretinal cysts in OS (Figure 1). There were also a few flame-shaped retinal hemorrhages and lipid exudates in both eyes at the initial presentation.

She was diagnosed with optic disc edema and SRD secondary to malignant hypertension (MHT), and intravenous pulse steroid therapy at the pregnancy dose (if not contraindicated) as well as regulation of blood pressure were recommended. For her headache, a cranial MRI was obtained, which was deemed normal. On the same day, placental rupture and intrauterine mort de fetus was diagnosed by the obstetrics and gynecology department, and she had an urgent cesarean section. During her hospitalization, the initial laboratory evaluation showed a lactate dehydrogenase (LDH) of 1233 U/L, aspartate aminotransferase (AST) of 51 U/L, alanine transaminase (ALT) of 21 U/L, platelet count of 53 x 109/L, activated partial thromboplastin time (APTT) of 23.5 sec, prothrombin time (PT) of 11.6 sec, and +3 protein in the spot urine test, consistent with pre-eclampsia with HELLP syndrome. The systemic blood pressure was regulated with an intravenous administration of a 4-gram loading dose of magnesium sulfate, followed by a maintenance dose of 1.5 grams per hour. Additionally, alpha-methyldopa was administered at a dose of 250 mg, three times a day. The patient, who was followed up in the intensive care unit for five days, was discharged on the eighth day after her vital signs and blood tests returned to normal.

**Figure 2:**
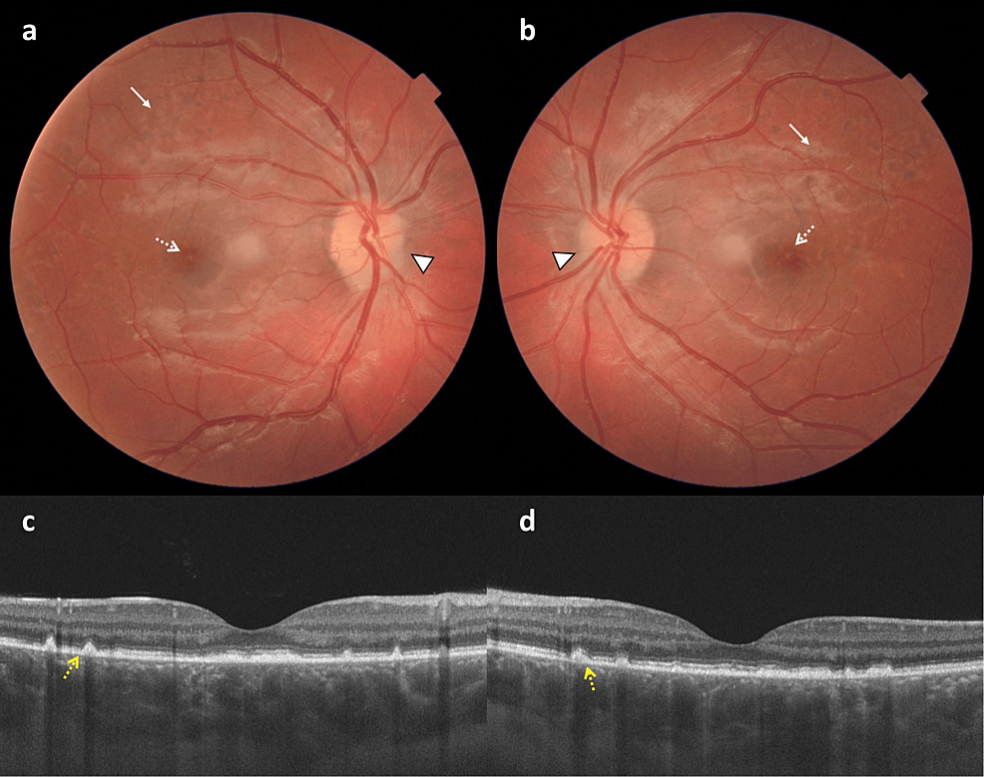
The color fundus photo and spectral domain optical coherence tomography scan at the first-month follow-up The color fundus picture shows subtle optic disc edema (white arrowhead), multiple dark, old choroidal infarct areas (white arrow) in the fundus periphery, and a drusen-like appearance in the macula (white dashed arrow) in the right (a) and left (b) eyes. Horizontal SD-OCT scan demonstrates subretinal drusenoid deposits (yellow dashed arrow) and disrupted ellipsoid zone in the macula, a thickened subfoveal choroid, and normal inner retinal layers in the right (c) and left (d) eyes. SD-OCT, spectral domain optical coherence tomography

Two weeks after her initial presentation, her BCVA had improved to 0.6 in both eyes with normal anterior segment findings. Slightly improved optic disc edema and mostly resolved subretinal fluid (SRF) were evident in both eyes. Her detailed ophthalmologic examination at the first-month follow-up showed normal pupillary reactions with no RAPD. Color vision assessed with the Ischiara chart was normal bilaterally. Anterior segment findings were unremarkable, and intraocular pressure was 15 mmHg in both eyes. Subtle optic disc edema, multiple dark, old choroidal infarct areas (Elschnig spots) in the fundus periphery, and a drusen-like appearance in the macula were prominent in fundoscopy bilaterally (Figure 2a and Figure 2b). The SD-OCT showed SDD in the macula, a thickened subfoveal choroid of 438 μm in OD and 443 μm in OS, and normal-looking inner retinal layers in both eyes (Figure 2c and Figure 2d).

Signed informed consent was obtained from the parents of the patient. 

## Discussion

Preeclampsia is a condition that often develops after the twentieth week of pregnancy and is characterized by increased blood pressure. HELLP syndrome, on the other hand, is a life-threatening pregnancy complication usually considered to be a variant of preeclampsia. It occurs in about 0.7% of pregnancies and affects nearly 15% of women with severe pre-eclampsia. So far, hypertensive changes of the retina and choroid, SRD, retinal vein occlusion, and cortical blindness have been shown to be related to HELLP syndrome [4]. Very recently, a few case reports of HELLP syndrome-related visual loss documented drusenoid deposits after the resolution of serous macular detachment [5,6].

Subretinal drusenoid deposits are a rare finding that has been associated with AMD. However, in recent years, SDDs have also been reported in patients with various other retinal diseases, including retinal dystrophies, vitamin A deficiency, congenital dyskeratosis, and pseudoxanthoma elasticum [7,8]. The exact pathogenesis of SDDs is still not fully understood. However, an increasing body of evidence from histopathological and OCT studies implicates choroidal vascular dysfunction in the pathogenesis [9]. Choroidal vascular dysfunction is a well-known finding of MHT, which is a component of HELLP syndrome [10]. However, several additional factors, including systemic inflammation, endothelial dysfunction, and microthrombi caused by disseminated intravascular coagulation seen in HELLP syndrome, may further exacerbate the choroidal ischemia and subsequent damage to the RPE and Bruch's membrane complex [11]. These alterations may also cause increased vascular permeability and fluid accumulation leading to SRD, which accompanied SDDs in previous case reports with HELLP syndrome, including ours [5,6]. In a cross-sectional study investigating the presence of SDD in patients with pre-eclampsia and malignant hypertension, Otero-Marquez et al. observed that SRD was present in all eyes with SDD [10]. The prevalence of SDD in pre-eclampsia and MHT patients was 32.7% and 23.4%, respectively, in this study. Multimodal imaging, including fluorescein angiography (FA), indocyanine green angiography (ICGA), and optical coherence tomography angiography (OCTA) documented impaired choroidal circulation in recent case reports diagnosed with HELLP syndrome [5,6].

Similar to our case, Van Rysselberge et al., reported hyperreflective deposits in the RPE/Bruch membrane complex in a 25-year-old patient with HELLP syndrome [6]. Those deposits, although reduced in number during the 1st year follow-up, were completely recovered at the final visit in the 4th year. This may be attributed to the recovery of choroidal circulation and the consequently improved health of the neighboring outer retina and RPE. The observation of the reperfusion of ischemic choroidal areas in OCTA slabs in a patient with HELLP syndrome after delivery and antihypertensive treatment may further support this theory [5].

Invasive imaging methods, including FA and ICGA, could not be performed on our patient due to her poor general health condition at the first presentation. Nevertheless, Elschnig spots seen in fundoscopy may be a sign of choroidal ischemia in our patient. Another limitation could be that our follow-up period was short due to the patient missing the control visits. As a result, we couldn't determine if the SDD lesions had regressed or not. Yet, to our knowledge, our case represents one of the youngest patients similar to those reported in the literature, along with the case documented by Zatreanu and Iyer [12]. A recent, large, population-based report by Curtin et al. showed that women with hypertensive disorders of pregnancy demonstrated an increased risk of neovascular AMD developing in later life [13]. This may be explained by the choroidal insufficiency shared by both entities. Therefore, retinal complications in pregnant women, including SDDs, may have significant long-term visual implications.

## Conclusions

In conclusion, the relationship between SDDs and preeclampsia or HELLP syndrome is not well-established, and further studies are needed to fully understand this association. Clinicians should be aware of the potential ocular complications associated with these hypertensive disorders and consider prompt ophthalmologic evaluation in affected patients.
